# Occurrence, Distribution, and Risk Assessment of Antibiotics in the Aquatic Environment of the Karst Plateau Wetland of Yangtze River Basin, Southwestern China

**DOI:** 10.3390/ijerph19127211

**Published:** 2022-06-12

**Authors:** Feng Guo, Yanan Wang, Jie Peng, Hetian Huang, Xiangting Tu, Hu Zhao, Nan Zhan, Zhu Rao, Gaofeng Zhao, Hongbo Yang

**Affiliations:** 1 Key Laboratory of Eco-Geochemistry, Ministry of Natural Resource, National Research Center for Geoanalysis, Beijing 100037, China; guofeng@mail.cgs.gov.cn (F.G.); wyn13985436504@126.com (Y.W.); 18798045936@163.com (J.P.); hetianhuang2022@163.com (H.H.); tuxiangtingtxt@163.com (X.T.); zhaohu21@mails.ucas.ac.cn (H.Z.); zhannan@mail.cgs.gov.cn (N.Z.); raozhu@126.com (Z.R.); 2 Key Laboratory of Environmental Pollution Monitoring and Disease Control, School of Public Health, Ministry of Education, Guizhou Medical University, Guiyang 550025, China; 3 Guiyang Public Health Clinical Center, Guiyang 550004, China; 4 Institute of Environment and Sustainable Development in Agriculture, Chinese Academy of Agricultural Sciences, Beijing 100081, China

**Keywords:** antibiotics, aquatic environment, plateau wetland, occurrence, distribution, risk assessment

## Abstract

In this study, the occurrence, distribution, and ecological risk of 40 commonly used antibiotics, including 15 sulfonamides (SAs), 9 fluoroquinolones (FQs), 7 macrolides (MCs), 3 tetracyclines (TCs), 2 chloramphenicols (CAPs), and 4 other categories, in the aquatic environment of the karst plateau wetland Caohai of the Yangtze River basin in southwestern China are reported. In total, 27 antibiotics were detected, with the detection rate ranging from 5% to 100%. The total concentration at each site ranged from 21.8 ng/L to 954 ng/L, with the average concentration being 189 ng/L. FQs and MCs were the most predominant categories, contributing 29.3% and 25.0% of the total antibiotic burden. The five most commonly detected antibiotics were ciprofloxacin (CIP), oxytetracycline (OTC), acetyl sulfamethoxazole (ASMZ), norfloxacin (NOR), and florfenicol (FF). The spatial distribution of the total concentration at each site demonstrated a decreasing trend from the southeastern area upstream adjoining the main counties to the northwestern area downstream, indicating that human activities have a great impact. Meanwhile, the natural attenuation rates of different types of antibiotics in the direction of flow ranged from 17.6% to 100%, which implied the natural purification potential of the wetland for antibiotics. The cluster analysis results indicated that domestic sewage and wastewater from agriculture and animal husbandry were the main sources of contamination in the surrounding wetland. Risk quotients (RQs) assessment showed that most of the individuals were at low to medium risk and that the adverse risks posed by mixtures of antibiotics were higher than those posed by the individual antibiotics.

## 1. Introduction

Antibiotics have been extensively used to treat infectious diseases and promote growth for both humans and animals since the advent of penicillin in 1929 [1]. At present, as a class of emerging organic pollutants, the resistance genes formed by antibiotics are listed as one of the three major threats to public health and the ecological environment by the World Health Organization [2]. It has been proven that antibiotics can induce drug resistance in pathogenic bacteria, which can accumulate in the environment and be transmitted to humans through the food chain, constituting a significant threat to human and animal health [3]. It has been reported that the consumption of antibiotics has increased from 54.1 billion standard units to 73.6 billion in 71 countries in the past ten years [4]. Most of these are incompletely absorbed in the target organisms and thereafter excreted as metabolites via feces and urine. The incomplete removal of waste in municipal sewage treatment plants as well as improper waste disposal can ultimately lead to antibiotics being released into the aquatic environment [5,6].The occurrence, migration, and transformation of antibiotics in the environment have attracted great attention in many environmental units, such as the surface water of rivers [7], sediment [8], soil [9], and even in groundwater [10]. It has been reported that the concentrations of 17 detected antibiotics ranged from ND to 544 ng/L in the Sine River of France [11]. Much higher concentrations in the range of 0.8–8464 ng/L were found in the surface water of Pakistan [12]. In China, the annual usage of antibiotics has been estimated to be 160,000 tons, with more than 50,000 tons being discharged into the environment, as reported in 2015 [13], which constitutes a huge environmental burden. The characteristics and levels of antibiotics in Chinese rivers and lakes, such as the Yellow River [14], the Pearl River [15], the Haihe River [16], the Huangpu River [17], Lake Chaohu [18], and Lake Taihu have been thoroughly studied [19]. The average concentration of antibiotics in the rivers of China is 303 ng/L, which is nearly three times higher than that of the United States and about 30 times higher than that of Italy [13]. Wetlands are special ecological units inhabited by a large number of aquatic animals and plants. They also possess many unique ecological functions, such as naturally purifying water [20], regulating runoff, as well as supplying groundwater. The existing research on this topic shows that wetlands are inevitably affected by human activities [21]; however, the understanding of the role played by antibiotics in the aquatic environment of wetlands disturbed by human activities is still limited. Specifically, determining the impact of antibiotics on wetlands is imperative. With the gradual development of pollution investigation, prevention, and control [22], it is necessary to gain more insight into the environmental prevalence and behavior of different types of antibiotics in wetlands to aid local risk control in the future.

Wetland Caohai is the source lake of Luoze River, a tributary of the Yangtze basin, which is one of the three largest plateau wetland systems situated on Yungui Plateau of Guizhou Province in the southwest of China [23]. It is a typical representative of the subtropical highland wetland ecosystem developed in this famous karst basin, with a mean depth of 1.35 m and a surface area of 96 km^2^. There are high levels of sunshine and abundant aquatic species in this wetland area, including 8 categories of algae and 38 species of aquatic vegetation. With the increase in population density and the proliferation of industry, agriculture, and animal husbandry in this area, adverse impacts on the wetland ecosystem are becoming apparent. Previous studies have reported the pollution of wetland Caohai by polycyclic aromatic hydrocarbons and heavy metals [24,25]. Limited basic data on the occurrence and behaviors of antibiotics in wetland Caohai are available. The objectives of the current paper are (i) to systematically investigate the occurrence and spatial distribution of the 40 selected antibiotics, including 15 sulfonamides (SAs), 9 fluoroquinolones (FQs), 7 macrolides (MCs), 3 tetracyclines (TCs), 2 chloramphenicols (CAPs), and 4 other antibiotics (monensin, salinomycin, lincomycin, cloxacillin), in the aquatic environment of wetland Caohai; (ii) to elucidate the spatial distribution, possible sources, and natural attenuation behaviors of different types of antibiotics in the studied wetland; and (iii) to evaluate the potential ecological risk posed by antibiotics to organisms. The results obtained provide an improved understanding and practical reference for the precise risk management and protection of wetland areas in the future.

## 2. Materials and Methods

### 2.1. Reagents and Chemicals

Thirty-eight antibiotics standards, including sulfamethazine (SMZ), sulfadiozine (SD), sulfacetamide (SCT), sulfapyridine (SPD), sulfamethoxazole (SMX), sulfadimethoxine (SSS), sulfathiazole (ST), acetylsulfamethoxazole (ASMZ), sulfisoxazole (SIZ), sufaguanidine (SG), sulfachloropyridazine (SCP), sulfameter (SM), sulfadoxine (SDO), sulfamerazine (SMR), sulfamonomethoxine (SMM), tertracycline (TC), oxytercycline (OTC), doxycycline (DC), marbofloxacin (MAR), fleroxacin (FLE), pefloxacine (PEF), norfloxacin (NOR), ciprofloxacine (CIP), danofloxacine (DAN), oflxacine (OFL), sarafloxacin (SAR), enroflxacine (ENR), roxithromycin (ROX), clarithromycin(CTM), oleanolomycin (ODM), leucomycin-A3 (LEU-A3), spiramycin (SPI), lincomycin (LIN), cloxacillin (CLOX), monensin (MON), salinomycin (SAL), chloramphenicol (CAP), and florfeniol (FF), were purchased from Dr. Ehrenstorfer GmbH (Augsburg, Germany). Tylosin (TYL) and erythromycin (ERY) were purchased from Sigma-Aldrich (St. Louis, MO, USA). Surrogate standards trimethoprim-d3 (TRI-d3), sulfamethazine-d4 (SMA-d4), sulfamethoxazole-d4 (SMX-d4), erythromycin-13C, d3 (ERY-13C, d3), lincomycin-d3 (LIN-d3), and thiabendazole-d4 (THI-d4) were obtained from Toronto Research Chemicals (Toronto, ON, Canada). Another two surrogate standards, ofloxacine-d3 (OFL-d3) and chloramphenicol-d5 (CAP-d5), were purchased from Sigma-Aldrich (St. Louis, MI, USA), Dr. Ehrenstorfer GmbH (Augsburg, Germany) individually. The basic chemical information of 40 antibiotics is shown in Table S1. All standards were dissolved in methanol and stored at 4 °C in the dark. HPLC-grade methanol, acetonitrile, and ammonium hydroxide were obtained from Fisher Scientific (Fair Lawn, NJ, USA). Formic acid (FA) was purchased from Tianjin Kermiou Chemical Reagent Co., Ltd. (Tianjin, China). Ethylenediaminetetraacetic acid disodium salt (Na_2_EDTA) was purchased from Sinopharm Group Chemical Reagent Co., Ltd. (Shanghai, China).

### 2.2. Sample Collection and Sample Preparation

**Figure 1:**
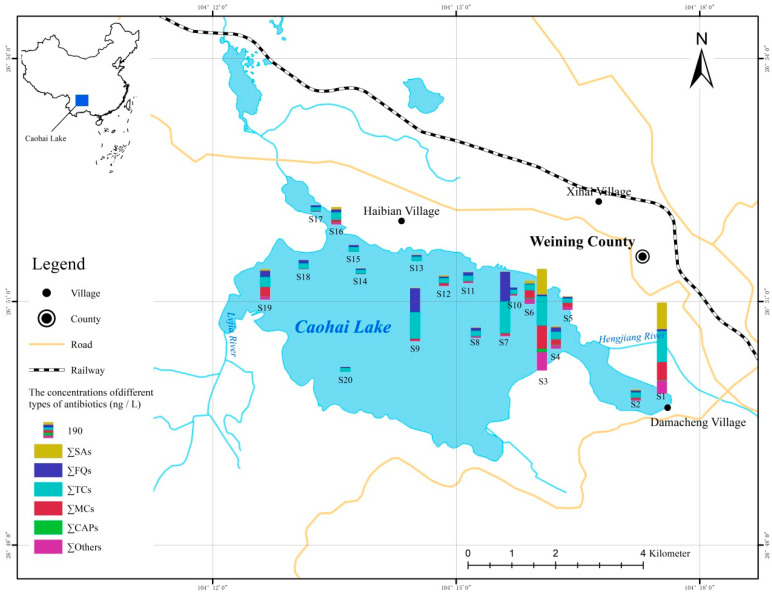
The sampling sites and spatial distribution of antibiotics in wetland Caohai.

According to the direction of flow and distribution of the sewage outlet, 20 surface water samples were collected from wetland Caohai in December 2017. These were divided into three groups: the upstream group (named Group 1), including S1 to S6; the center area group (named Group 2), including S7–S12 and S20; and the downstream group(named Group 3), including S13–S19. The sampling sites are shown in Figure 1. Samples approximately 0–50 cm below the water’s surface were collected in pre-cleaned amber glass bottles (1.0 L) and stored below 4 °C to prevent degradation. All the samples were extracted within one week. 

An aliquot of 500 mL of water samples was filtered through a 0.45 μm microporous membrane. The filtered water was adjusted to pH = 4 with formic acid and then 20 ng surrogate standards and 0.25 g Na_2_EDTA were added. Water samples were loaded at a flow rate of 5.0 mL/min to Waters Oasis HLB cartridges (500 mg, 6 mL), which were preconditioned sequentially with 6.0 mL of methanol and 6.0 mL of ultra-pure water. After sample loading, the cartridges were rinsed with 12.0 mL of ultra-pure water and dried under a vacuum. The targets retained on the cartridges were eluted with 6.0 mL of ammonia–methanol (5:95, *v*/*v*) solution, and then, the eluent was evaporated to near dryness under a gentle stream of nitrogen and reconstituted in 0.5 mL of methanol–water (1:9, *v*/*v*). Finally, the extract was centrifuged at 12,000 r/min for 10 min. The supernatants were transferred to a 2.0 mL amber vial and stored at 4.0 °C until analysis.

### 2.3. Instrumental Analysis

The analysis of antibiotics was performed on an Agilent 1260 liquid chromatograph coupled with an Agilent 6460 triple quadrupole MS equipped with an electrospray ionization (ESI) source (Agilent, Palo Alto, Santa Clara, CA, USA). The optimum mass spectrum parameters for the antibiotics are shown in Table S2. Separation was achieved using an Agilent Zorbax Rrhd Eclipse Plus C18 column (2.1 mm × 50 mm i.d., 1.8 μm). The column temperature was maintained at 40 °C. The flow rate was kept at 0.2 mL/min and the injection volume was 5.0 μL. The analysis was performed in negative mode for two target compounds (FF, CAP) and in positive mode for the other compounds. For negative mode, the mobile phase consisted of eluent A (ultra-pure water) and B (methanol–acetonitrile; 1:1, *v*/*v*). The separation of FF and CAP was achieved with the following gradient program: 0.0–2.0 min, 25.0–60.0% B; 2.0–4.0 min, 60.0–80.0% B; 4.0–5.0 min, 100% B; 5.0–7.0 min, 100% B; 7–7.01 min, 100–25.0% B; and 7.01–9.0 min 25.0% B. For positive mode, the mobile phase consisted of eluent A (0.2% formic acid and 2 mM ammonium acetate) and B (methanol–acetonitrile; 1:1, *v*/*v*). The separation of the other antibiotics was achieved with the following gradient program: 0–0.5 min, 5% B; 0.5–5.0 min, 5–20.0% B; 5.0–10.0 min, 20.0–40.0% B; 10.0–14.0 min, 40.0–70.0% B; 14.0–17.0 min, 70.0–100% B; 17.0–20.0 min, 100% B; 20.0–20.01 min, 100–5.0% B; and 20.01–23.0 min, 5% B.

### 2.4. Quality Control

The concentrations of the target compounds in all the samples were determined using the internal standard method. The performance of the method was satisfying for target antibiotics within the linearity range of 1.0 ng/L to 200 ng/L and with correlation coefficient *r*^2^ ranging from 0.991 to 0.999. The limits of detection (LODs) and the limits of quantification (LOQs) were determined as the concentrations corresponding to signal-to-noise (S/N) ratios of 3 and 10. The LODs and the LOQs of antibiotics in water samples ranged from 0.002 to 0.270 ng/L and ranged from 0.007 to 0.900 ng/L, respectively. The recoveries of the antibiotics spiked to 500 mL surface water samples (*n* = 3) ranging from 61.0 to 149%. The relative standard deviations (RSDs, %) in duplicated samples (*n* = 3) ranged from 0.19% to 32.0%, values which were comparable with those found in previous studies. More information is shown in detail in Table S3. The procedural blank was run in parallel every 10 samples to check for laboratory contamination; the concentrations of all target compounds were below the detection limit. Check standards were run every 10 samples in sequence to check the system performance.

### 2.5. Statistical Analysis

Statistical analyses were performed with IBM PASW Statistics 17.0. The cluster analysis was used for the common sources of multiple antibiotics as suggested. The affinity relationship between detected antibiotics is measured by similarity coefficient under the similarity of 30%.

Ecological risk was assessed by Risk Quotient (RQ) and Mixtured Risk Quotient (MRQ). The formula was as follows:

(1) $$\mathrm{RQ}=\frac{MEC}{PNEC}$$

(2) $$\mathrm{PNEC}=\frac{LC50\;\mathrm{or}\;EC50}{AF}$$

(3) $$\mathrm{MRQ}=\sum_{i=1}^{n}RQi$$

where *MEC* is the measured environmental concentration; PNEC is the predicted no-effect concentration; *LC*50 or *EC*50 are the short-time toxicity data; and *AF* is the assessment factor.

## 3. Results and Discussion

### 3.1. The Occurrence Characteristics of Antibiotics in the Wetland Caohai

**Figure 2:**
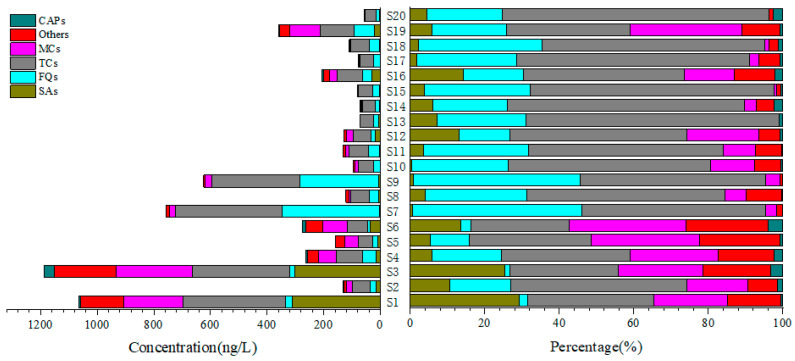
Detected concentration and composition profiles of antibiotics at all sample sites in wetland Caohai.

**Figure 3:**
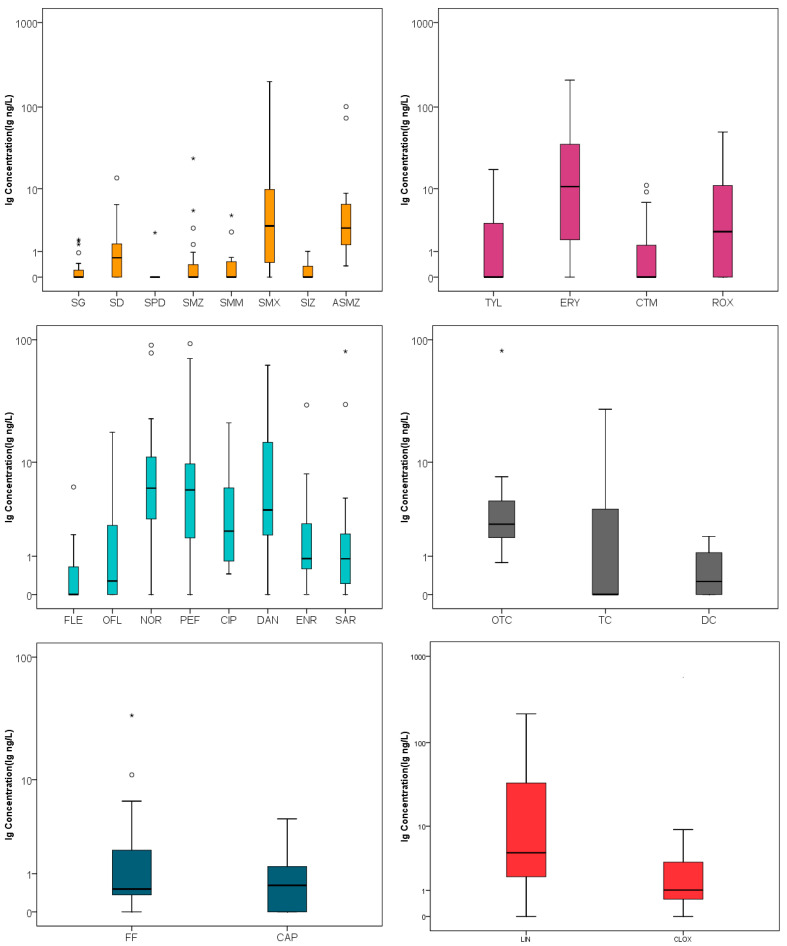
The concentration characteristics of different antibiotics in wetland Caohai (“○” represents outliers more than 1.5 quartiles away from the quartiles, and “*” represents outliers more than 3 quartiles away from the quartiles. Thin lines at the top and bottom of the box indicate the maximum and minimum values excluding outliers.).

A total of 27 of 40 antibiotics were detected, while SCT, ST, SMR, SM, SCP, SDO, SSS, MAR, SPI, LEU-A3, ODM, SAL, and MON were not detected in any sample. As shown in Table S4, 27 antibiotics were detected at more than half of the sampling sites. The top five individual antibiotics found were CIP, OTC, ASMZ, NOR, and FF. CIP and OTC were the most frequently detected individuals, with a detection rate of 100%. ASMZ, NOR, and FF were the second most predominant compounds, with a detection rate of 95%, followed by PEF and DAN with a detection rate of 90%, all of which indicated the widespread presence of antibiotics in wetland Caohai. In terms of categories, the total concentrations of FQs ranged from ND to 93.0 ng/L, with the highest average concentration of 55.1 ng/L found among all categories, followed by MCs (ND-209 ng/L, 47.0 ng/L), SAs (ND-201 ng/L, 39.6 ng/L), others (ND-216 ng/L, 31.7 ng/L), TCs (ND-81.8 ng/L, 10.7 ng/L), and CAPs (ND-34.2 ng/L, 4.2 ng/L). All samples showed very similar composition profiles (seen Figure 2), which demonstrated that FQs made up 29.3% of the total antibiotic burden, followed by MCs (25.0%), SAs (21.0%), others (16.8%), TCs (5.70%), and CAPs (2.20%). Detection rate greater than 70% and average concentrations greater than 3.0 ng/L in the aquatic environment of wetland Caohai were compared with the values found in related studies, as shown in Table 1. Generally speaking, the concentration of antibiotic residues in Caohai was found to be at medium level in this study. The concentration characteristics of different types of individual antibiotics in wetland Caohai are shown in Figure 3, and the details are provided as follows.

FQs were found to be the most predominant antibiotics in Caohai, which is consistent with the findings obtained for Yancheng coastal wetlands [34] but different from those obtained for Baiyangdian Lake [35]. Among the investigated FQs, the detection rate of individuals ranged from 40% to 100%, with most of them being greater than 50%. CIP showed the highest detection rate of 100%, and only MAR was not detected in any samples. Individuals with the highest detection rate were not necessarily detected in the highest concentration. The concentration range of each individual was PEF (ND-93.0 ng/L) > NOR (ND-90.5 ng/L) > SAR (ND-80.8 ng/L) > DAN (ND-62.9 ng/L) > ENR (ND-30.0 ng/L) > CIP (0.50–21.5 ng/L) > OFL (ND-17.9 ng/L) > FLE (ND-6.00 ng/L). The highest value obtained for SAR in this area was much higher than the value of 28.2 ng/L obtained for Baiyangdian Lake [35], the value of 15.6 ng/L obtained for Taihu Lake [27], the value of 35.9 ng/L obtained for Haihe river [16], and the value of 10.0 ng/L obtained for the Seine River [11] although this substance was removed from clinical use by its manufacturer Abbott Laboratories on 30 April 2001. This implies the wide usage of this antibiotic in the studied area and means that it should be paid more attention to, due to its severe adverse effects on organisms. The highest concentration of PEF detected in this study was far lower than the value detected in Taihu Lake [27], which was 323 ng/L. A similar concentration range of NOR was found to that of Chaohu Lake in China [18], but this range was far higher than that of the Po River in Italy (1.83–2.39 ng/L) [31]. FQs have been reported to be widely used in livestock husbandry, especially for pigs and chickens [13]. 

MCs were the second most common type of antibiotic found in Caohai. The detection rate of the individual antibiotics ranged from 40% to 80%. ROX and ERY were the most frequently detected, with detection rates of 80.0% and 70.0%, respectively. These are known to be the most common macrolide antibiotics used for humans, and SPI, LEU-A3, and ODM were not found. The concentration of ROX was in the range of ND-50.3 ng/L, which was similar to the concentration found in the Weihe river (1.57–59.5) [33] in China and far lower than that found for the Liaohe river (ND-741ng/L) [26], the Pearl river (ND-169 ng/L) [15], and the Pakistan basin (ND-183 ng/L) [12]. For ERY, the highest concentration found was 209 ng/L, and it was regularly found in municipal wastewater treatment plants [36].

The proportion of SAs was comparable to that of MCs, with the detection rate ranging from 5% to 95.0%. Almost half of the targeted antibiotics, including SCT, ST, SMR, SM, SCP, SDO, and SSS, were not detected. SMX, ASMZ, and SD were the most frequently detected individual antibiotics. It has been reported that SMX and ASMZ often show a similar detection rate, since ASMZ is known to be the metabolite of SMX [37]. The concentrations of SMX and SD were in the ranges of ND-201 ng/L and ND-13.7 ng/L, respectively, which were similar to those of the Weihe river in China [38]. The concentration of SMX was much higher than that of Taihu Lake, Dongting Lake [39], and Bosten Lake [30]. In addition to the wide usage of this antibiotic for both humans and animals [40], the high detection rate and concentration of SMX might be due to its stronger migration ability and better biological stability than other sulfonamide antibiotics [41].

For TCs, the detection rates of OTC, DC, and TC were 100%, 50%, and 40%, respectively. They were obtained at relatively lower concentrations of 0.80–81.8 ng/L, ND-1.9 ng/L, and ND-27.8 ng/L, respectively, which might be due to their strange adsorption ability in sediment [42] as well as their strong hydrolysis characteristics [43].

FF is known as one of the top five most used veterinary antibiotics for pigs and chickens in China [13] and was detected at a high rate of 95.0% in Caohai, with a concentration range of ND-34.2 ng/L. Regarding CAP, although its use has been banned in the animal and husbandry and aquaculture industries due to its strong toxicity [44], it was still detected with a relatively high detection rate of 60.0%, which would justify its stronger regulation even if it was detected at a low maximum concentration of 4.40 ng/L. 

LIN has been reported as one of the most commonly used antibiotics for both humans and animals in China [13]. It was detected in a high concentration of ND-216 ng/L and had the highest detection rate of 85.0%; this was followed by CLOX, with a high concentration of ND-9.0 ng/L and a detection rate of 80.0%. However, MON and SAL were not detected in any samples, although they are known as feed additives and animal-specific antibiotics.

### 3.2. The Cluster Analysis and Potential Sources of Antibiotics in Wetland Caohai

In order to trace the common sources of multiple antibiotics in the aquatic environment of Caohai, a cluster analysis was performed. The affinity relationship between detected antibiotics is measured by similarity. As shown in the Figure S1, under the similarity of 40%, it can be divided into four categories. As shown in Figure S1, the first category consisted of SCT, SPI, SAL, CAP, SMR, ERY, SDO, LEU-A3, LIN, SM, CTM, SMX, ASMZ, SMM, SPD, OTC, SMZ, MAR, MON, FF, SCP, ROX, SD and TC. The second one consisted of ST, TYL, SSS, ODM, CLOX, FLE, SAR NOR, PEF, CIP, DAN and DC, the third one consisted of OFL and ENR, the fourth one consisted of SIZ. It is known that individuals in the same category are correlated with each other, which implies that they might come from the same pollution source. For the first category, SCT, SPI, CAP, SDO, LEU-A3, SM, CTM, SMM, SPD, SMZ, SMX, ASMZ, ERY, ROX and TC are commonly used to treat eye, urinary tract, lung and intestinal tract infections caused by bacteria or viruses [45,46] and thus likely have a human source [47,48]. SAL, SMR, MAR, OTC, SD, LIN, MON, FF and SCP are known or have been reported as antibiotics typical used in veterinary applications in previous studies [13,19,49,50]. Wetland Caohai is located on the southeast estuary of Caohai, with villages and farmlands concentrated nearby and about 109,000 people in surrounding Weining County. Composting human or animal manure is known to be one of the major modes of agricultural production in this county; thus, the antibiotics in the category 1 may be attributed to waste from residential or agricultural areas, such as domestic sewage and agricultural wastewater [24]. For the second category, most of the individuals are known or have been reported as antibiotics typical used in veterinary [51,52]. which have also been frequently detected at high levels in animal wastewater [33,36] as well as in environment [5]. The second category indicated that, except for human emissions, the scattered livestock or poultry breeding that takes place in this area might be another potential source of antibiotics in wetland Caohai. OFL, ENR and SIZ in the third and fourth category revealed similar sources of human and livestock mentioned above, for example, ENR and OFL have been regularly reported to be excreted and can enter the aquatic environment via the direct discharge of wastewater [53,54]. In summary, the domestic sewage and wastewater from agricultural and animal husbandry were the main sources of contamination in the surrounding wetland.

### 3.3. The Spatial Distribution and Attenuation Behavior of Antibiotics in Wetland Caohai

The total concentrations of the antibiotics detected at 20 sampling sites in wetland Caohai ranged from 21.8 ng/L to 954 ng/L, with the average concentration being 189 ng/L, as seen in Figure 2. The variation coefficient of the total concentration of antibiotics detected at each sampling point was 129%, which demonstrated the obvious difference in the spatial distribution of the antibiotic concentration. The highest total concentration was found at S3 of the upstream entrance adjacent to Weining County. At this point, MCs, TCs, CAPs, and others were detected at relatively high levels of 270 ng/L, 111 ng/L, 37.8 ng/L, and 216 ng/L, respectively, which indicated the intensive interference of human activities, as discussed in Section 3.2. The lowest total concentration was found at S20, located in the southwest region of wetland Caohai, which is relatively far away from Weining County. According to the grouping mentioned in Section 2.2, the average concentration was 374 ng/L in the southeast upstream area, followed by 155 ng/L in the central area and 50.7 ng/L in the northwest downstream area, which showed an obvious decreasing trend from the southeast upstream area to the northwest downstream area (see Figure 1). Thus, the average attenuation rate in the direction of flow was 86.4%. From the perspective of the individual antibiotics, different antibiotic individuals showed different attenuation behaviors in the aquatic environment of wetland Caohai. In detail, the average concentration of 27 detected individuals showed a downward trend from upstream to downstream, and their attenuation rate differed from 17.6% to 100% (seen in Table S4). Among these, the attenuation rate of most SAs, TCs, and MCs was more than 75%. These results reflect the natural ability of wetlands such as wetland Caohai to remove antibiotics, which is consistent with the previous studies on the ShiJiuyang wetland in Jiaxing City [55] and the Jiaozhou Bay wetland of China [56]. On the other hand, the concentration of seven detected antibiotics in the downstream area was higher than that in the upstream area; four individuals of these belonged to FQs, which might because the FQs had a stable nitrogen double-ring structure and a longer half-life period than that of the TCs, SAs, and MCs [57]. Furthermore, the high adsorption coefficient values (*K_d_*) of FQs also make them prone to be adsorbed in organic matter and thus accumulate in wetland areas [58,59]. The increasing levels of these seven individual antibiotics seen in the downstream area also indicate the existence of point sources in the direction of flow. It is worth noting that the total concentrations at the two sample sites S7 and S9 in the central area of wetland Caohai were higher than those of the surrounding area; this might because the total organic carbon (TOC) in the center of wetlands is generally higher than that of the surroundings. Additionally, the total concentration of antibiotics was reported to be positively correlated with TOC in a previous study [60].

### 3.4. Risk Assessment of Antibiotics in Wetland Caohai

The potential ecological risk posed by the detected antibiotics to aquatic organisms was assessed due to the wide detection of these substances and their proven adverse effects. Risk quotient (RQ) is the most commonly used measure for predicting the ecological risk posed by environmental pollutants [61,62]; it is calculated as a ratio of the measured environmental concentration (MEC) to the predicted no-effect concentration (PNEC) [34] based on the values previously reported in the literature. If PNEC cannot be obtained, it can be calculated by dividing the toxicity data, such as LC50 or EC50, by the assessment factor (AF). Generally, the AF of short-term toxicity data is 1000, while that of long-term toxicity data is 100; these values are listed in Table S5. 

**Figure 4:**
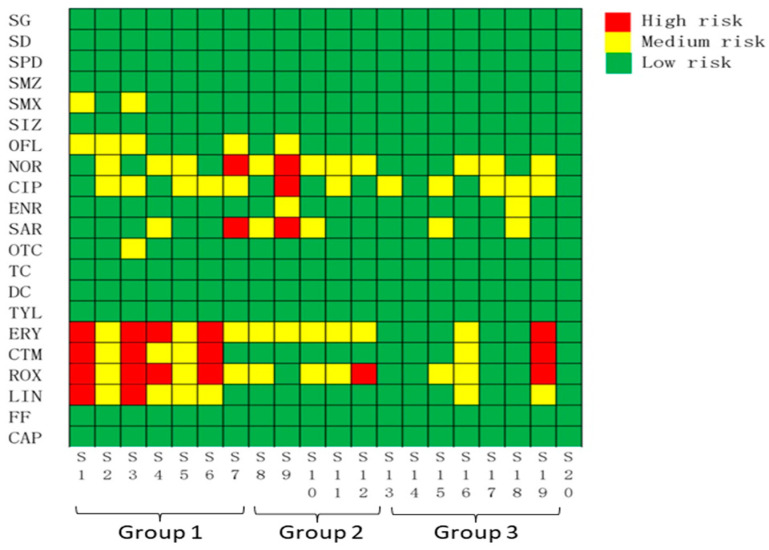
The risk based on RQs calculated for antibiotics in the aquatic environment of wetland Caohai. (The details of Group 1, Group 2 and Group 3 were defined in Table S7).

Algae has been proven to be the most sensitive species to antibiotics in the aquatic environment [63,64,65]. Based on this, the PNECs of algae were selected for use in the calculation of RQ. When RQ < 0.1, this indicates that there is a low risk to aquatic organisms in the study area; a value of 0.1 ≤ RQ < 1 suggests that there is a medium risk; and a value of RQ ≥ 1.0 reveals that there is a high risk. The calculated PNECs are presented in Table S5. The RQs calculated for algae are listed in Table S6 and shown in Figure 4. NOR, CIP, SAR, ERY, CTM, ROX, and LIN showed a high level of threat to organisms in the aquatic environment of wetland Caohai, with the maximum calculated RQs of 1.87, 1.26, 5.39, 5.71, 5.53, 10.9, and 3.09, respectively; these were distributed across S3, S7, and S9. SMX, OFL, ENR, and OTC showed a medium level of threat to organisms, with RQs ranging from 0.131 to 0.854. The RQs of SG, SD, SPD, SMZ, SIZ, TC, DC, TYL, FF, and CAP were all less than 0.10, which indicated that these antibiotics posed a slight risk to aquatic organisms in Caohai. One third of the detected antibiotics showed a high risk to aquatic species, which were distributed in 40% of the sampling sites where there was at least one antibiotic deemed to constitute a high risk. On the spatial scale, the sites at high risk were concentrated in the southeastern and middle regions of Caohai, which are adjacent to the residential and agricultural areas. 

The coexistence of multiple antibiotics in the ecosystem of wetland Caohai might enhance the ecological risk via the cocktail effect [66,67,68]. The classical mixture toxicity concentration addition (CA) model was used to calculate the risk quotients of the mixtures (MRQ) [69], which are the sum of the RQs for detected individuals. The results are shown in Table S7. The MRQ values were in the range of 0.13–26.5 at all the sampling sites, and were obviously higher than those found by individual assessments. Except for S13, S14, S15, S17, S18, and S20, the MRQ values of all sampling sites were higher than 1, suggesting that these sampling sites were at relatively high ecological risk and accounting for 70% of the samples. These were much higher compared to those assessed using the RQ value of the individual antibiotics, which indicated that the adverse effects of the antibiotic mixtures on aquatic species were higher than those of individual antibiotics. Given lack of toxicity data for SMM, ASMZ, FLE, PEF, DAN, and CLOX, no RQs were included even when they were detected at levels of up to 4.3 ng/L, 101 ng/L, 6.00 ng/L, 93.0 ng/L, 62.8 ng/L, and 9.00 ng/L. More toxicology data and further risk assessments are required due to the potential threat posed by antibiotic-resistant genes to the ecological system and human health [70]. 

## 4. Conclusions

This study represents a rare effort to systematically investigate the occurrence, distribution, and ecological risk posed by a wide range of commonly used antibiotics in the aquatic environment of the karst plateau wetland Caohai in southwest China. The target antibiotics were widely detected, even those whose use is now forbidden. The average concentration of antibiotics in wetland Caohai was at a medium level compared with the values found in other studies, such as those on the Seine river and the Hai river. The decreasing trend found for the total average concentration of antibiotics from upstream to downstream indicates the natural ability of wetlands to remove antibiotics. The attenuation rate of different types of antibiotics varied greatly and more fundamental data, such as data on the main factors controlling hydrochemistry for the purification potential evaluation of wetlands, are necessary, since these were determined jointly by chemical and biological stability, regional use, and environmental capacity. A preliminary judgement of the domestic sewage and agricultural and animal husbandry sources of the surrounding wetland was made. The adverse effect of the mixture of antibiotics on aquatic species in the area was shown to be worse than that of the individual antibiotics. This study provides a practical reference for the precise risk management of the aquatic environment in wetland Caohai. Long-term monitoring, land source investigation, and more toxicology data on antibiotics are required in the future to further improve risk control. 

## Figures and Tables

**Table 1:** Comparison of the concentration of antibiotics in the aquatic environment in related studies (ng/L).

Study Area	SMX	NOR	PEF	DAN	SAR	OTC	ROX	ERY	LIN	Reference
This study	ND-201	ND-90.5	ND-93	ND-62.8	ND-80.8	0.80–81.8	ND-50.3	ND-209	ND-216	–
The Yellow River	<LOQ-56	<LOQ-300	–	–	–	–	<LOQ-95.0	<LOQ-77.0	–	[14]
The Liao River	ND-670	ND-256	–	–	–	ND-742	ND-741	ND-2834	–	[26]
The Pearl river	<LOQ-193	<LOQ-251	–	–	–	–	<LOQ-169	ND-30.6	–	[15]
The Huangpu River	2.2–765	ND	–	–	–	11.5–84.5	0.2–4.1	–	–	[17]
The Hai River	ND-201	ND-141	–	–	ND-35.9	–	ND-40.2	ND-67.7	–	[16]
Taihu Lake	ND-234	ND-12.2	ND-323	ND-34.1	ND-15.6	ND-34.8	ND-35.6	ND-4.66	ND-53.8	[27]
Chaohu Lake	ND-171.6	ND-70.2	–	–	–	ND-2.90	–	–	–	[28,18]
Baiyangdian Lake	ND-940	ND-1140	–	–	ND-28.2	4.28–90.3	ND-302	ND-121	–	[29]
Bosten Lake	1.12–13.3	–	–	–	–	ND-20.7	–	–	–	[30]
Po River	1.83–2.39	–	–	–	–	ND-1.82	–	0.28–4.62	–	[31]
Pakistan part region	<LOQ-2700	<LOQ-38	–	–	–	1.10–1100	<LOQ-180	<LOQ-310	<LOQ-1100	[12]
Seine river	ND-53.0	ND-163	–	–	–	–	–	–	–	[11]
The Yong jiang River	<LOQ-68.0	–	–	–	–	–	ND-6.10	ND-174	–	[32]
The Wei he River	7.60–115	ND-39.2	–	–	–	ND-104	1.57–59.5	23.3–59.5	3.63–125	[33]

“–” Not detected.

## Supplementary Materials

The following supporting information can be downloaded at: https://www.mdpi.com/article/10.3390/ijerph19127211/s1, Table S1: The basic chemical information of 40 antibiotics in this study; Table S2: The Optimum mass spectrum parameters for the antibiotics; Table S3: The limits of detection, recoveries, calibration curve and correlation coefficient of the method; Table S4: The concentration, detection rate and spatial coefficient of variation of antibiotics in the aquatic environment of the wetland Caohai; Table S5: Toxicity data of 21 antibiotics to algae; Table S6: The RQs of each sampling point at Caohai wetland; Table S7: MRQ for the antibiotics in wetland Caohai; Figure S1: The cluster analysis of the detected antibiotics in wetland Caohai. References [26,71,72,73,74,75,76,77,78,79] are cited in the supplementary materials.
