# Supplementary material for: Occurrence, Distribution, and Risk Assessment of Antibiotics in the Aquatic Environment of the Karst Plateau Wetland of Yangtze River Basin, Southwestern China
Source: Int J Environ Res Public Health. 2022 Jun 12;19(12):7211. doi: 10.3390/ijerph19127211 (PMC9223651; doi:10.3390/ijerph19127211)
Supplement: Supplementary file 1 [file ijerph-19-07211-s001.zip › ijerph-1712067-supplementary.pdf]

Table S1 The basic chemical information of 40 antibiotics in this study

Types	Analytes	CAS	Abbreviation	Chemical formula	Molecular Weight	Chemical Structure
sulfonamides	sufaguanidine	57-67-0	SG	$C_7H_{10}N_4O_2S$	214.2	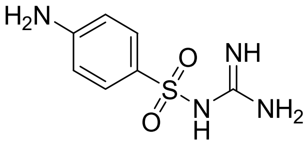
	sulfacetamide	144-80-9	SCT	$C_8H_{10}N_2O_3S$	214.2	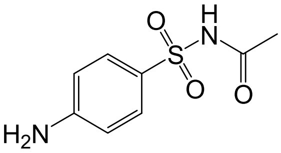
	sulfadiazine	68-35-9	SD	$C_{10}H_{10}N_4O_2S$	250.3	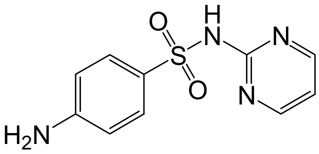
	sulfathiazole	72-14-0	ST	$C_9H_9N_3O_2S_2$	255.3	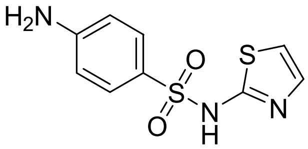
	sulfapyridine	144-83-2	SPD	$C_{11}H_{11}N_3O_2S$	249.3	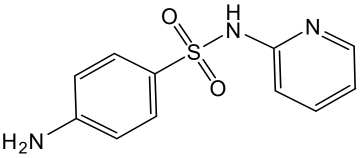
	sulfamerazine	127-79-7	SMR	$C_{11}H_{11}N_4NaO_2S$	286.3	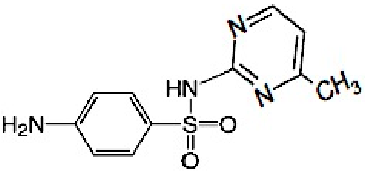
	sulfameter	651-06-9	SM	$C_{11}H_{12}N_4O_3S$	280.3	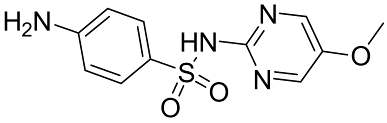
	sulfamethazine	57-68-1	SMZ	$C_{12}H_{14}N_4O_2S$	278.3	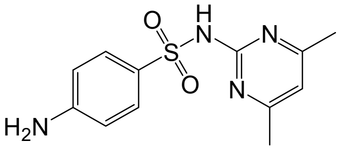
	sulfachloropyridazine	80-32-0	SCP	$C_{10}H_9ClN_4O_2S$	284.7	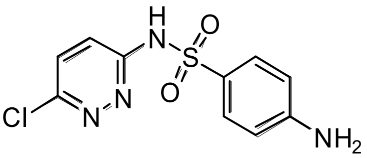

	sulfamonomethoxine	1220-83-3	SMM	$C_{11}H_{12}N_4O_3S$	280.3	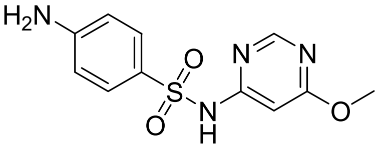
	sulfamethoxazole	723-46-6	SMX	$C_{10}H_{11}N_3O_3S$	253.3	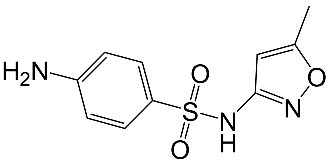
	sulfisoxazole	127-69-5	SIZ	$C_{11}H_{13}N_3O_3S$	267.3	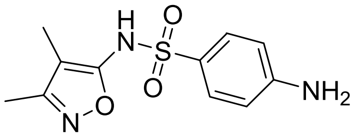
	acetylsulfamethoxazole	21312-10-7	ASMZ	$C_{12}H_{13}N_3O_4S$	295.3	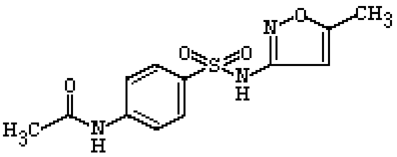
	sulfadoxine	2447-57-6	SDO	$C_{12}H_{14}N_4O_4S$	310.3	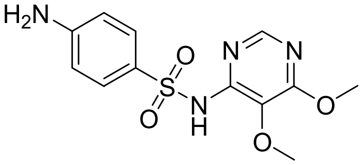
	sulfadimethoxine	122-11-2	SSS	$C_{12}H_{14}N_4O_4S$	310.3	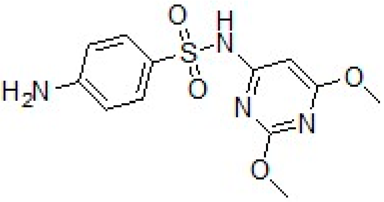
Fluoroquinolones	marbofloxacin	115550-35-1	MAR	$C_{17}H_{19}FN_4O_4$	362.4	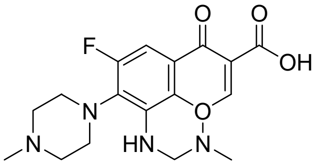
	fleroxacin	79660-72-3	FLE	$C_{17}H_{18}F_3N_3O_3$	369.34	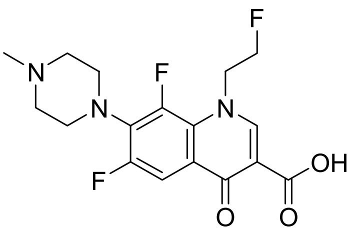
	oflxacine	82419-36-1	OFL	$C_{18}H_{20}FN_3O_4$	361.3	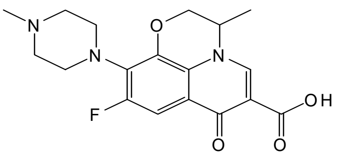

	norfloxacin	70458-96-7	NOR	$C_{16}H_{18}FN_3O_3$	319.3	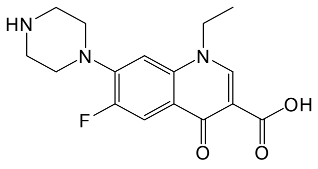
	pefloxacine	70458-92-3	PEF	$C_{17}H_{20}FN_3O_3$	333.4	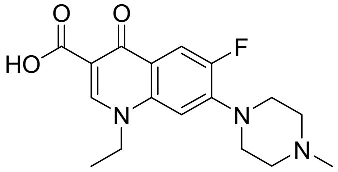
	ciprofloxacine	85721-33-1	CIP	$C_{17}H_{18}FN_3O_3$	331.3	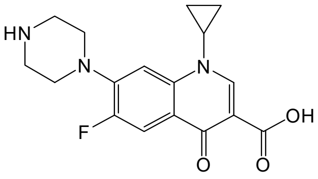
	danofloxacine	112398-08-0	DAN	$C_{19}H_{20}FN_3O_3$	357.4	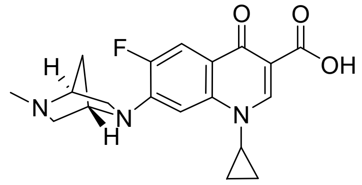
	enroflxacine	93106-60-6	ENR	$C_{19}H_{22}FN_3O_3$	359.4	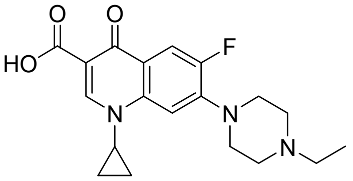
	sarafloxacin	98105-99-8	SAR	$C_{20}H_{17}F_2N_3O_3$	385.4	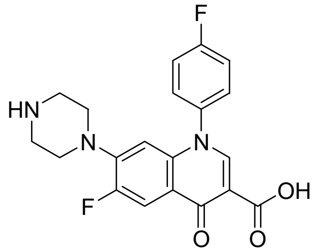
tetracyclines	oxytercycline	79-57-2	OTC	$C_{22}H_{24}N_2O_8$	460.4	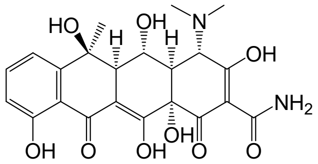
	tertracycline	60-54-8	TC	$C_{22}H_{24}N_2O_8$	444.4	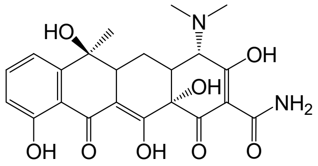

	doxycycline	564-25-0	DC	$C_{22}H_{24}N_2O_8$	444.4	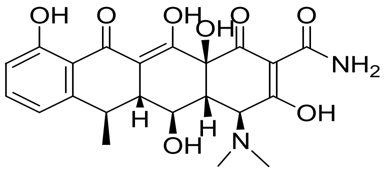
macrolides	spiramycin	8025-81-8	SPI	$C_{43}H_{74}N_2O_{14}$	842.4	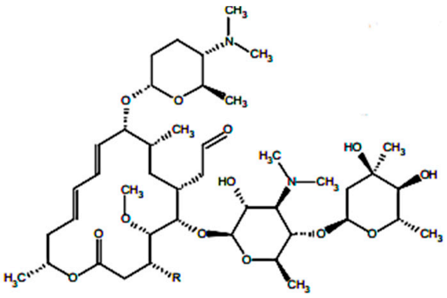
	tylosin	1401-69-0	TYL	$C_{46}H_{77}NO_{17}$	916.1	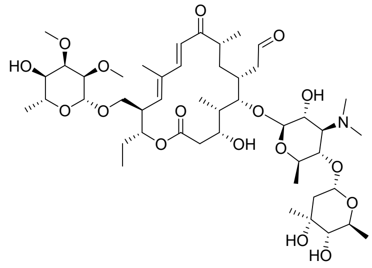
	erythromycin	114-07-8	ERY	$C_{37}H_{67}NO_{13}$	733.9	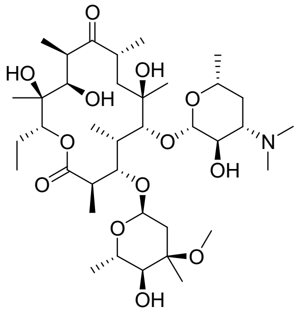
	clarithromycin	81103-11-9	CTM	$C_{38}H_{69}NO_{13}$	748.0	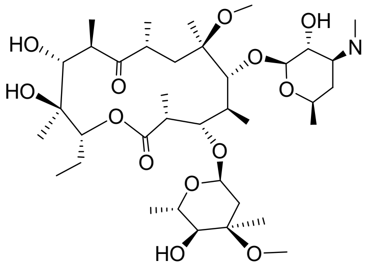
	roxithromycin	80214-83-1	ROX	$C_{41}H_{76}N_2O_{15}$	837.0	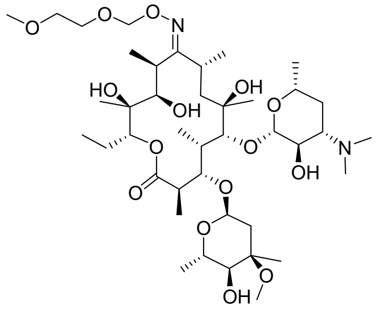

	leucomycin A3	16846-24-5	LEU-A3	$C_{42}H_{69}NO_{15}$	828.0	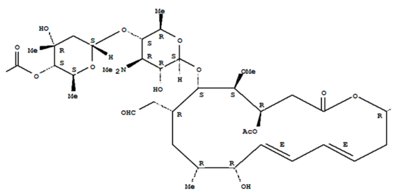
	oleandomycin	3922-90-5	ODM	$C_{35}H_{61}NO_{12}$	687.9	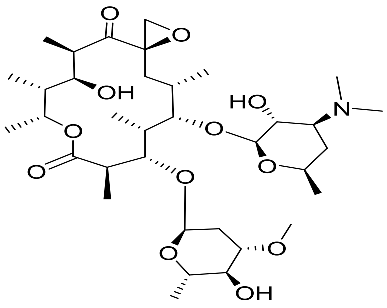
chloramphenicols	florfenicol	73231-34-2	FF	$C_{12}H_{14}Cl_2FNO_4S$	358.2	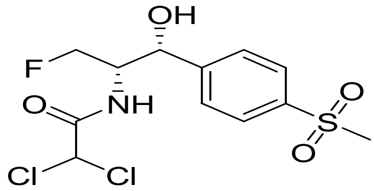
	chloramphenicol	154-75-2	CAP	$C_{11}H_{12}Cl_2FN_2O_5$	323.1	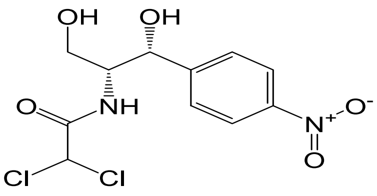
others	monensin	17090-79-8	MON	$C_{36}H_{62}O_{11}$	670.9	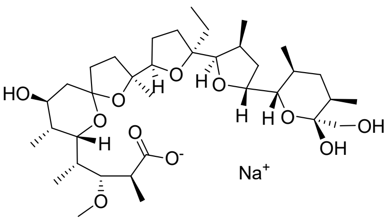
	salinomycin	53003-10-4	SAL	$C_{42}H_{70}O_{11}$	751	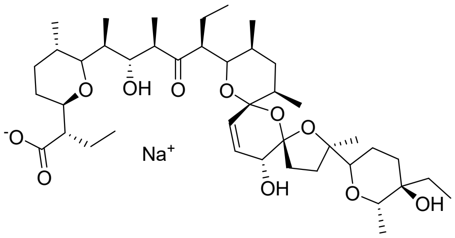
	lincomycin	154-21-2	LIN	$C_{18}H_{34}N_2O_6S$	406.5	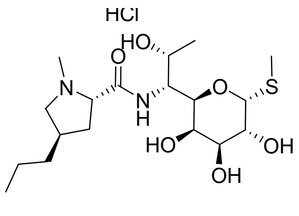

cloxacillin	61-72-3	CLOX	$C_{19}H_{18}ClN_3O_5S$	435.9	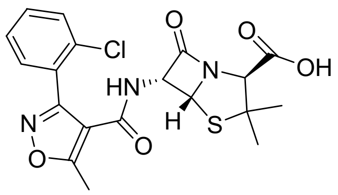

Table S2 The Optimum mass spectrum parameters for the antibiotics

Analytes	Retention Time (min)	Precursorion (m/z)	Production (m/z)	Fragmentor (V)	Collision energy (eV)
SG	2.545	215.1	92.2*, 64.9	81	24, 44
SCT	1.057	215.1	156*, 92.2	72	24, 8
SD	3.655	251.1	92.2*, 156.1	107	24, 16
ST	4.746	256.2	156.1*, 92.2	106	11, 24
SPD	5.121	250.1	92.3*, 156.1	100	24, 14
SMR	5.621	265.1	91.9*, 156	91	24, 14
SM	7.128	281.2	91.9*, 108.1	100	34, 24
SMZ	7.183	279.2	186.3*, 124.2	106	14, 24
SCP	8.413	285.2	92.2*, 64.9	81	24, 44
SMM	8.508	281.2	91.9*, 155.8	121	34, 15
SMX	8.943	254.4	156*, 108.1	90	13, 21
SIZ	9.746	268.2	156.1*, 113	96	8, 14
ASMZ	10.513	296.4	133.9*, 73.2	106	19, 24
SDO	11.409	311.1	156.3*, 92.1	103	17, 26
SSS	9.44	311.1	156.3*, 92.2	112	18, 32
MAR	7.157	363.2	72.2*, 69.9	114	25, 37
FLE	7.449	370.0	326.2*, 269.1	145	20, 28
OFL	7.729	362.2	318.5*, 261.5	114	17, 25
NOR	7.771	320.2	302.2*, 275.6	123	17, 19
PEF	7.826	334.1	316.1*, 293.2	131	18, 11
CIP	8.064	332.2	314*, 288	124	17, 17
DAN	8.562	358.2	340*, 283.1	165	17, 20
ENR	8.69	360.2	342.4*, 316.3	119	19, 17
SAR	9.405	386.3	368.3*, 342.2	120	19, 17
OTC	7.653	461.2	426.5*, 201.1	119	17, 41
TC	7.974	445.2	410.4*, 428.6	111	17, 15
DC	11.246	445.2	428.1*, 154	134	17, 25
SPI	10.988	843.6	174*, 144.8	150	41, 36
TYL	14.373	917.1	173.8*, 100.5	190	41, 57
ERY	14.67	716.5	158.2*, 558	160	25, 25
CTM	15.2	748.9	158.1*, 590	180	24,16
ROX	15.371	838.1	158.1*, 680.8	150	23, 19
LEU-A3	15.466	828.6	109.1*, 174.1	191	44, 36
ODM	13.198	689.4	158.2*, 545.9	127	34, 14
FF	3.037	356	336.1*, 185.1	-90	-4, -16
CAP	3.787	356	152.1*,413.4	-100	-13, -56
MON	20.12	693.4	461.6*, 675.9	170	60, 40
SAL	20.426	773,3	431.3*, 413.4	160	55, 56
LIN	6.315	407.5	126.1*, 70.1	155	32, 72

CLOX	15.286	436.2	178.2*, 220.3	173	24, 14
SMA-D4	7.109	283.1	124.1*, 186.1	109	24, 16
SMX-D4	8.885	258.1	160.1*, 112	110	13, 21
LIN-D3	6.317	410.6	129.1*, 73.1	170	36, 76
THI-D4	6.315	206.1	179.1*, 135.1	130	25, 35
TRI-D4	6.855	294.4	123*, 230	152	21, 21
OFL-D3	7.714	366.3	322.5*, 261.6	103	18, 30
ERY-13C,D3	15.228	720.6	162.2*, 562.2	162	25, 20
CAP-D5	3.762	326.1	157.1*, 261.8	-110	-14, -6

* The transitions were used for quantification.

Table S3 The limits of detection, recoveries, calibration curve and correlation coefficient of the method

Analytes	Linearity Range （ng/mL）	LODs (ng/L)	LOQs (ng/L)	Spiked Concentration and Precision						Calibration curve	r^2	Internal standdard
				5 (ng/L)	RSD (%) (n = 3)	50 (ng/L)	RSD (%) (n = 3)	100 (ng/L)	RSD (%) (n = 3)			
SG	0.50 - 200	0.058	0.193	74.5	5.55	72.8	4.09	72.7	3.66	Y=0.496804X-0.001702	0.998	SMA-D4
SCT	0.50 - 200	0.048	0.160	71.5	11.8	62.9	6.6	62.1	3.57	Y=0.603164X-0.001988	0.998	SMA-D4
SD	0.50 - 200	0.074	0.247	100	18.5	73.9	6.31	71.3	5.39	Y=0.46659X-0.0006870	0.991	SMA-D4
ST	0.50 - 200	0.057	0.190	105	25.8	87.5	19.8	109	25.2	Y=0.322985X-0.002166	0.996	SMA-D4
SPD	0.50 - 200	0.033	0.110	80.8	16.9	77.1	0.69	78.2	1.3	Y=0.564652X+0.001209	0.998	SMA-D4
SMR	0.50 - 200	0.034	0.113	85.3	8.8	84.7	4.45	85.5	2.54	Y=0.333182X+0.000269	0.998	SMA-D4
SM	0.50 - 200	0.042	0.140	99.2	1.23	102	20.1	104	1.8	Y=0.527594X+0.009430	0.99	SMA-D4
SMZ	0.50 - 200	0.024	0.080	90.5	9.56	122	0.75	93.2	3.18	Y=0.499592X+0.003572	0.998	SMA-D4
SCP	1.00 - 200	0.107	0.357	90.6	12.9	93.5	2.39	85.4	4.69	Y=0.1694X-0.000837894	0.998	SMA-D4
SMM	0.50 - 200	0.056	0.187	107	18.6	79.9	17.9	127	12.7	Y=0.00538X+0.094610	0.998	SMA-D4
SMX	0.50 - 200	0.069	0.230	120	9.71	93.6	15	77	6.79	Y=0.175726X-0.001203	0.997	SMX-D4
SIZ	0.50 - 200	0.08	0.267	83.3	15.3	68.5	14.2	68.5	19.1	Y=0.192927X-0.003782	0.992	SMA-D4
ASMZ	1.00 - 200	0.079	0.260	95.6	10.9	68.5	9.67	117	3.69	Y=0.043806X-0.0005174	0.995	SMA-D4
SDO	0.50 - 200	0.034	0.113	69	4.24	94.7	3.85	69	2.56	Y=1.119341X+0.009763	0.996	SMA-D4
SSS	0.50 - 200	0.035	0.117	68	3.36	101	19	67.6	2.56	Y=0.014237X+0.12235	0.999	SMA-D4
MAR	1.00 - 200	0.060	0.200	79	8.04	99.7	7.62	62.9	10.3	Y=3.806642X+0.020986	0.993	OFL-D3
FLE	1.00 - 200	0.230	0.767	66.2	13.1	82.4	13.1	72.7	6.26	Y=5.094687X-0.034279	0.999	OFL-D3
OFL	0.50 - 200	0.164	0.547	83.4	8.08	96.6	21.3	100	4.57	Y=4.910041X-0.028751	0.999	OFL-D3
NOR	1.00 - 200	0.143	0.477	66	9.75	75.8	14.7	80.8	8.3	Y=0.470729X-0,033099	0.992	OFL-D3
PEF	1.00 - 200	0.250	0.833	71.2	2.46	80.3	3.13	149	13.9	Y=4.482062X-0.033436	0.999	SAR-D8
CIP	1.00 - 200	0.133	0.443	64	19.4	102	8.54	77.9	2.72	Y=2.828346X-0.043408	0.994	OFL-D3
DAN	1.00 - 200	0.182	0.607	99.3	23.4	75.7	10.2	125	2.28	Y=1.488973X-0.013613	0.994	SAR-D8
ENR	1.00 - 200	0.160	0.533	84.1	5.26	130	6.67	80.3	6.93	Y=5.142616X-0.057320	0.998	OFL-D3
SAR	1.00 - 200	0.270	0.900	88.7	9.87	73.7	5.06	97.4	10	Y=5.458254X-0.069007	0.997	SAR-D8
OTC	1.00 - 200	0.160	0.533	61	3.15	124	20.3	98.7	7.77	Y=0.029048X-0.000234	0.994	TRI-D3
TC	1.00 - 200	0.032	0.107	77	16.1	111	21.8	72.9	14.1	Y=0.040243X+0.000210	0.992	TRI-D3
DC	1.00 - 200	0.147	0.490	96.1	14.9	92.2	2.75	83.6	10.5	Y=0.076058X-0.0007741	0.995	TRI-D3
SPI	1.00 - 200	0.021	0.070	103	14.9	95.1	0.95	83.6	10.5	Y=0.011336X-0.000160	0.995	ERY-13 C-D3
TYL	1.00 - 200	0.007	0.023	100	8.34	90.5	0.95	111	16.3	Y=0.042834X-0.000428	0.999	ERY-13 C-D3
ERY	1.00 - 200	0.025	0.0803	131	13.5	118	18	128	9.12	Y=0.062230X+0.000064	0.998	ERY-13 C-D3
CTM	1.00 - 200	0.023	0.077	129	21.6	116	20.8	93	12.1	Y=1.030182X+1.322590	0.998	ERY-13 C-D3
ROX	1.00 - 200	0.033	0.110	119	9.48	109	10	67.9	13.9	Y=0.074442X-0.000179	0.999	ERY-13

												C-D3
LEU-A3	0.50 - 200	0.008	0.027	104	14.2	67.3	28.9	110	12.2	Y=0.768968X+2.179457	0.998	ERY-13 C-D3
ODM	0.50 - 200	0.015	0.050	76.7	3.52	120	14.1	86.5	13.4	Y=0.434659X+0.001054	0.997	ERY-13 C-D3
FF	0.50 - 200	0.002	0.007	96.7	13.3	90.9	7.45	83.7	6.02	Y=1.664007X+0.001819	0.999	CAP-D5
CAP	0.50 - 200	0.055	0.183	93.1	14.9	83.9	27	79	2.97	Y=0.867775X+0.001819	0.999	CAP-D5
MON	0.50 - 200	0.011	0.037	94	8.3	94.5	10.3	93.3	13.3	Y=0.139559X+0.000242	0.999	THI-D4
SAL	1.00 - 200	0.151	0.503	82.2	21	74.6	5.53	81.8	8.36	Y=0.153363X+0.000929	0.996	THI-D4
LIN	0.50 - 200	0.018	0.060	126	32	86.5	5.76	91.7	10.9	Y=0.565537X-0.0000820	0.999	LIN-D3
CLOX	1.00 - 200	0.134	0.447	72.2	19.2	78.3	4.09	81.4	10.8	Y=0.047691X-0.0000675	0.998	THI-D4

Table S4 The concentration, detection rate and spatial coefficient of variation of antibiotics in the aquatic environment of the wetland Caohai

Compound	Frequency(%)	Concentration (ng/L)				The attenuation rate*（%）	LogKOW
		Median value	Minimum value	Maximum value	Average value		
Sufaguanidine (SG)	25.0	ND	0.45	1.80	0.30	-100	-1.0716
Sulfacetamide (SCT)	0.00	ND	0.00	ND	ND	-	-0.6044
Sulfadiazine (SD)	70.0	0.70	0.24	13.7	1.80	-88.0	-0.3377
Sulfathiazole (ST)	0.00	ND	0.00	ND	ND	-	0.7151
Sulfapyridine (SPD)	5.00	ND	2.33	2.30	0.10	-100	0.5266
Sulfamerazine (SMR)	0.00	ND	0.00	ND	ND	-	0.2096
Sulfameter (SM)	0.00	ND	0.00	ND	ND	-	-0.2568
Sulfamethazine (SMZ)	25.0	ND	0.96	24.0	1.70	-95.5	0.7569
Sulfachloropyridazine (SCP)	0.00	ND	0.00	ND	ND	-	0.3068
Sulfamonomethoxine (SMM)	35.0	ND	0.00	4.30	0.50	-93.7	0.1981
Sulfamethoxazole (SMX)	80.0	3.00	0.39	201	23.3	-95.1	0.484
Sulfisoxazole (SIZ)	40.0	ND	0.23	1.00	0.20	63.1	1.0313
Acetylsulfamethoxazole(ASMZ)	95.0	2.80	0.36	101	11.7	-90.9	1.2077
Sulfadoxine (SDO)	0.00	ND	0.00	ND	ND	-	-0.2378
Sulfadimethoxine (SSS)	0.00	ND	0.00	ND	ND	-	1.1745
Marbofloxacin (MAR)	0.00	ND	0.00	ND	ND	-	0.57620
Fleroxacin (FLE)	40.0	ND	0.32	6.00	0.60	-	0.0463
Oflxacine (OFL)	50.0	0.30	0.63	17.9	2.80	-90.6	-0.1998
Norfloxacin (NOR)	95.0	5.90	1.39	90.5	14.9	-21.1	-0.306
Pefloxacine (PEF)	90.0	5.70	1.35	93.0	12.8	113	-0.0948
Ciprofloxacine (CIP)	100	2.20	0.45	21.5	4.30	-17.6	-0.0008
Danofloxacine (DAN)	90.0	3.60	0.55	62.8	10.0	85.8	0.4421
Enroflxacine (ENR)	80.0	0.90	0.59	30.0	3.00	139	0.7015

Sarafloxacin (SAR)	75.0	0.90	0.49	80.8	6.60	68.3	1.0713
Oxytercycline (OTC)	100	2.60	0.79	81.8	7.00	-79.7	-2.8669
Tertracycline (TC)	40.0	ND	3.09	27.8	3.20	-77.2	-1.3286
Doxycycline (DC)	50.0	0.30	0.60	1.90	0.60	79.3	-1.3645
Spiramycin (SPI)	0.00	ND	0.00	ND	ND	-	1.8735
Tylosin (TYL)	40.0	ND	1.07	17.5	2.90	-44.4	1.0496
Erythromycin (ERY)	80.0	10.7	1.36	209	34.6	-95.6	4.3405
Clarithromycin (CTM)	40.0	ND	0.47	11.1	1.70	-95.9	3.1776
Roxithromycin (ROX)	70.0	2.40	0.60	50.3	7.80	-95.9	2.7508
Leucomycin A3 (LEU-A3)	0.00	ND	0.00	ND	ND	-	3.1591
Oleanolomycin (ODM)	0.00	ND	0.00	ND	ND		1.8319
Florfeniol (FF)	95.0	0.50	0.00	34.2	3.30	-89.8	-0.0365
Chloramphenicol (CAP)	60.0	0.60	0.00	4.40	0.90	-49.8	0.916
Monensin(MON)	0.00	ND	0.00	ND	ND	-	5.4325
Salinomycin(SAL)	0.00	ND	0.46	ND	ND	-	8.5263
Lincomycin(LIN)	85.0	4.40	0.22	216	29.5	94.3	0.2884
Cloxacillin(CLOX)	80.0	1.00	0.17	9.00	2.20	70	3.2181

The attenuation rate(%) was calculated by（$C_{Group\ 3}$-C_{Group1}）/C_{Group1}*100%, C_{Group1} and $C_{Group\ 3}$ represents the average total concentration of antibiotics in Group 1 and Group 3, respectively. “-” represents the average total concentration of antibiotics in Group 3 was lower than that of Group 1.

Table S5 Toxicity data of 21 antibiotics to algae

antibiotics	Tested species	Toxicity data / (mg/L)	Toxicity type	Assessment factor	PNEC/(ng/L)	Reference
SMX	selenastrum capricornutum	1.53	acute	1000	1530	[71]
SD	selenastrum capricornutum	2.19	acute	1000	2190	[71]
OTC	selenastrum capricornutum	0.342	acute	1000	342	[72]

ERY	selenastrum capricornutum	0.0366	acute	1000	36.6	[72]
TYL	selenastrum capricornutum	0.411	acute	1000	411	[71]
CIP	M.aeruginosa	0.017	acute	1000	17	[73]
OFL	M.aeruginosa	0.021	acute	1000	21	[73]
ENR	M.aeruginosa	0.049	acute	1000	49	[74]
LIN	Pseudokichneriella subcapitata	0.07	acute	1000	70	[72]
CTM	Pseudokichneriella subcapitata	0.002	acute	1000	2	[72]
TC	Pseudokichneriella subcapitata	3.31	acute	1000	3310	[75]
SMZ	S.vacuolatus	19.52	acute	1000	19520	[73]
SPD	Lemna minor	0.46	acute	1000	460	[73]
NOR	Microcystis aeruginosa	0.0485	acute	1000	48.5	[26]
ROX	Green algae	0.0046	acute	1000	4.6	[76]
FF	chlorella pyenoidosa	12.3	acute	1000	12300	[77]
CAP	chlorella pyenoidosa	28.66	acute	1000	28660	[77]
SAR	Microcystis aeruginosa	0.015	acute	1000	15	[78]
SG	Scenedesmus vacuolatus	3.42	acute	1000	3420	[73]
SIZ	Scenedesmus vacuolatus	11.9	acute	1000	11900	[73]
DC	Lemna gibba	1.844	acute	1000	1844	[79]

Table S6 The RQs of each sampling point at Caohai wetland

Compounds	SG	SD	SPD	SMZ	SMX	SIZ	0FL	NOR	CIP	ENR	SAR	OTC	TC	DC	TYL	ERY	CTM	ROX	LIN	FF	CAP
S1	0.00	0.00	0.00	0.00	0.13	0.00	0.75	0.00	0.09	0.05	0.00	0.01	0.00	0.00	0.00	5.06	4.54	3.58	2.16	0.00	0.00
S2	0.00	0.00	0.00	0.00	0.00	0.00	0.27	0.13	0.23	0.00	0.08	0.02	0.00	0.00	0.00	0.44	0.31	0.83	0.15	0.00	0.00
S3	0.00	0.01	0.01	0.00	0.12	0.00	0.31	0.03	0.39	0.00	0.06	0.24	0.01	0.00	0.00	5.71	5.53	10.94	3.09	0.00	0.00
S4	0.00	0.00	0.00	0.00	0.00	0.00	0.03	0.44	0.06	0.05	0.14	0.00	0.00	0.00	0.01	1.23	0.98	2.57	0.55	0.00	0.00
S5	0.00	0.00	0.00	0.00	0.00	0.00	0.00	0.15	0.16	0.02	0.00	0.00	0.00	0.00	0.04	0.77	0.24	0.50	0.46	0.00	0.00
S6	0.00	0.00	0.00	0.00	0.01	0.00	0.00	0.05	0.12	0.01	0.03	0.01	0.00	0.00	0.00	1.74	1.64	4.26	0.84	0.00	0.00
S7	0.00	0.00	0.00	0.00	0.00	0.00	0.15	1.62	0.96	0.07	5.39	0.00	0.00	0.00	0.02	0.32	0.00	0.56	0.05	0.00	0.00
S8	0.00	0.00	0.00	0.00	0.00	0.00	0.07	0.19	0.08	0.07	0.32	0.01	0.00	0.00	0.00	0.17	0.00	0.14	0.04	0.00	0.00
S9	0.00	0.00	0.00	0.00	0.00	0.00	0.85	1.87	1.26	0.61	2.02	0.00	0.00	0.00	0.04	0.16	0.00	0.00	0.06	0.00	0.00
S10	0.00	0.00	0.00	0.00	0.00	0.00	0.00	0.19	0.03	0.00	0.11	0.02	0.00	0.00	0.00	0.21	0.00	0.77	0.06	0.00	0.00
S11	0.00	0.00	0.00	0.00	0.00	0.00	0.00	0.27	0.31	0.02	0.05	0.01	0.00	0.00	0.00	0.26	0.00	0.38	0.06	0.00	0.00
S12	0.00	0.00	0.00	0.00	0.01	0.00	0.00	0.11	0.03	0.01	0.04	0.01	0.00	0.00	0.00	0.40	0.00	2.22	0.04	0.00	0.00
S13	0.00	0.00	0.00	0.00	0.00	0.00	0.00	0.03	0.46	0.02	0.00	0.00	0.00	0.00	0.00	0.00	0.00	0.00	0.00	0.00	0.00
S14	0.00	0.00	0.00	0.00	0.00	0.00	0.00	0.05	0.04	0.01	0.00	0.02	0.00	0.00	0.00	0.06	0.00	0.00	0.03	0.00	0.00

S15	0.00	0.00	0.00	0.00	0.00	0.00	0.00	0.09	0.11	0.01	0.20	0.01	0.00	0.00	0.00	0.00	0.00	0.13	0.00	0.00	0.00
S16	0.00	0.00	0.00	0.00	0.01	0.00	0.06	0.11	0.03	0.01	0.05	0.01	0.00	0.00	0.02	0.45	0.45	0.64	0.29	0.00	0.00
S17	0.00	0.00	0.00	0.00	0.00	0.00	0.05	0.18	0.15	0.04	0.06	0.01	0.00	0.00	0.00	0.00	0.00	0.00	0.00	0.00	0.00
S18	0.00	0.00	0.00	0.00	0.00	0.00	0.00	0.09	0.39	0.16	0.12	0.01	0.00	0.00	0.00	0.04	0.00	0.00	0.02	0.00	0.00
S19	0.00	0.00	0.00	0.00	0.01	0.00	0.09	0.48	0.14	0.06	0.09	0.01	0.00	0.00	0.01	1.90	3.29	6.31	0.50	0.00	0.00
S20	0.00	0.00	0.00	0.00	0.00	0.00	0.00	0.08	0.04	0.00	0.00	0.01	0.00	0.00	0.00	0.00	0.00	0.00	0.00	0.00	0.00
RQ_{sum}	16.3	2.45	26.4	6.07	2.34	8.72	9.13	1.09	6.89	1.41	1.37	2.86	0.51	0.22	0.55	2.11	0.50	0.83	12.9	0.13	103

Table S7 MRQ for the antibiotics in wetland Caohai

Sampling points	MRQ	Group*
S1	16.4	1
S2	2.46	1
S3	26.4	1
S4	6.07	1
S5	2.34	1
S6	8.72	1
S7	9.13	2
S8	1.09	2
S9	6.89	2
S10	1.41	2
S11	1.37	2
S12	2.86	2
S13	0.51	3
S14	0.22	3
S15	0.55	3

S16	2.11	3
S17	0.49	3
S18	0.82	3
S19	12.9	3
S20	0.12	2

*It was in traduced in the main text Part 2.2. Here Group 1 represents “Upstream of Wetland Caohai”; Group 2 represents “Medium stream of Wetland Caohai”; Group 3 represents “Downstream of Wetland Caohai”.

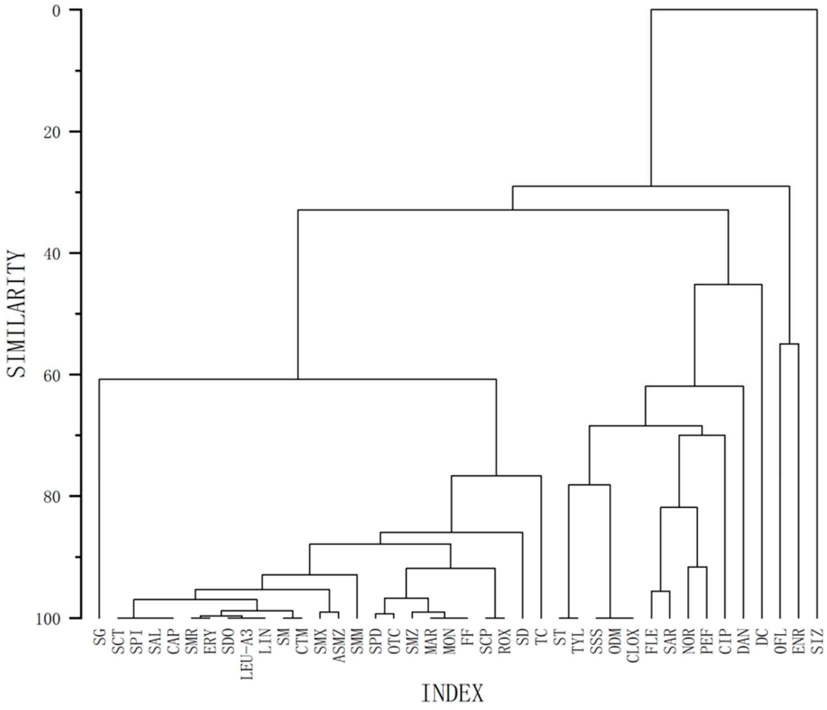

Figure S1 The cluster analysis of the detected antibiotics in wetland Caohai

Reference

Bialk, Bielinska., Stolte, S., Arning, J., Uebers, U., Boschen, A., Stepnowski, P., Matzke, M., 2011. Ecotoxicity evaluation of selected sulfonamides. Chemosphere., 85(6):928-933.

Brain, R.A., Johnson, D.J., Richards, S.M., Sanderson, H., Sibley, P.K., Solomon, K.R., 2004. Effects of 25 pharmaceutical compounds to Lemna gibba using a seven-day static-renewal test. Environmental Toxicology & Chemistry., 23(2):371-382.

H. C. Holten Lützhft., Halling,S.R.B., Rgensen, S.E., 1999. Algal Toxicity of Antibacterial Agents Applied in Danish Fish Farming. Archives of environmental contamination and toxicology., 36(1):1-6.

Eguchi, K., Nagase, H., Ozawa, M., Endoh, Y.S., Goto, K., Hirata, K., Yoshimura, H., 2004. Evaluation of antimicrobial agents for veterinary use in the ecotoxicity test using microalgae., Chemosphere., 57(11):1733-1738.

Gao, L.H., Shi, Y.L., Li, W.H., Liu, J.M., Cai, Y.Q., 2012. Occurrence, distribution and bioaccumulation of antibiotics in the Haihe River in China. Journal of Environmental Monitoring.,14(4):1248-1256.

Isidori, M., Lavorgna, M., Nardelli, A., Pascarella, L., Parrella, A., 2005. Toxic and genotoxic evaluation of six antibiotics on non-target organisms. Science of the Total Environment., 346(1-3):87-98.

Jiang, L., Hu, X., Yin, D., Zhang, H., Yu, Z., 2011. Occurrence, distribution and seasonal variation of antibiotics in the Huangpu River, Shanghai, China. Chemosphere., 82(6):822-828.

Li, X., Chen, J.F., Lv, S.H., Chen, J.Y., 2012. Growth inhibition efect of phenicol antibiotics on Chlorellapyenoidosa. Journal of Jinan university., 33(3):294-299.

Luo, L.C., Qin, B.Q., 2008. Hydrodynamics and Its Effects on the Aquatic Ecosystem. Lake Taihu, China. 2008.

Lei, X., Lu, J., Liu, Z., Tong, Y., Li, S., 2015. Concentration and distribution of antibiotics in water-sediment system of Bosten Lake, Xinjiang. Environmental

science and pollution research international., 22(3):1670-1678.

Robinson, A.A., Belden, J.B., Lydy, M.J., 2005. Toxicity of fluoroquinolone antibiotics to aquatic organisms. Environmental Toxicology and Chemistry., 24(2):423-430.

Tamtam, F., Mercier, F., Lebot, B, Eurin, J., Tuc, D.Q., Clement, M., Chevreuil M., 2008 Occurrence and fate of antibiotics in the Seine River in various hydrological conditions. Science of the Total Environment., 393(1):84-95.

Tang, J., Shi, T., Wu, X., Cao, H., Li, X., Hua, R., Yue, Y., 2015. The occurrence and distribution of antibiotics in Lake Chaohu, China: seasonal variation, potential source and risk assessment. Chemosphere., 122:154-161.

Watanabe, N., Bergamaschi, B.A., Loftin, K.A., Meyer, M.T., Harter, T., 2010. Use and environmental occurrence of antibiotics in freestall dairy farms with manured forage fields. Environmental Science & Technology., 44(17):6591-6600.

Wei, H., Wang, J.W., Yang, X.Y., Sun, B.C., Li, K.B., Zhang, J.T., 2017. Contamination characteristic and ecological risk of antibiotics in surface water of the Weihe Guanzhong section. China Environmental Science, 37(6):2255-2262.

Xu, W.H., Zhang, G., Zou, S.C., Li, X., Liu, Y., 2007. Determination of selected antibiotics in the Victoria Harbour and the Pearl River, South China using high-performance liquid chromatography-electrospray ionization tandem mass spectrometry. Environmental Pollution, 145(3):672-679.

Xu, W, Zhang, G, Zou, S., Ling, Z., Wang, G., Yan, W., 2009. A preliminary investigation on the occurrence and distribution of antibiotics in the Yellow River and its tributaries, China. Water Environment Research., 81(3):248-254.

Xue, B., Zhang, R., Wang, Y., Liu, X., Li, J., Zhang, G., 2013. Antibiotic contamination in a typical developing city in south China: Occurrence and ecological risks in the Yongjiang River impacted by tributary discharge and anthropogenic activities. Ecotoxicol Environ Saf., 92(3):229-236.

Yang, L.H., Ying, G.G., Su, H.C., Stauber, J.L., Aams, M.S., Binet, M.T., 2008. Growth-inhibiting effects of 12 antibacterial agents and their mixtures on the

freshwater microalga Pseudokirchneriella subcapitata. Environmental Toxicology & Chemistry., 27(5):1201-1208.

Zhang, Q., Xin, Q., Zhu, J.M., Cheng, J.P., 2013. The antibiotic contaminations in the main water bodies in China and the associated environmental and human health impacts. Environmental chemistry., 33(7):1075-1083.

Zhang, X.J., Bo, Y.W., Zhang, Y., Ma, S.Q., Guo, C.S., Zhang, L., 2017. Occurrence，distribution，and ecological risk of antibiotics in surface water in the Liaohe River Basin，China. Environmental Science, 38(11):4553-4561.

Zhou, L.J., Wu, Q.L., Zhao, Y.G., Zhang, B.B., 2016. Occurrence, spatiotemporal distribution, mass balance and ecological risks of antibiotics in the subtropical shallow Lake Taihu, China. Environmental Science: Processes and Impacts, 18(4):500-513.

Zuccato, E., Castiglioni, S., Bagnati, R., Melis, M., Fanelli, R., 2010. Source, occurrence and fate of antibiotics in the Italian aquatic environment. Journal of Hazardous Materials., 179(1):1042-1048.
